# Optimization of Traced Neuron Skeleton Using Lasso-Based Model

**DOI:** 10.3389/fnana.2019.00018

**Published:** 2019-02-21

**Authors:** Shiwei Li, Tingwei Quan, Cheng Xu, Qing Huang, Hongtao Kang, Yijun Chen, Anan Li, Ling Fu, Qingming Luo, Hui Gong, Shaoqun Zeng

**Affiliations:** ^1^Britton Chance Center for Biomedical Photonics, Wuhan National Laboratory for Optoelectronics-Huazhong University of Science and Technology, Hubei, China; ^2^MoE Key Laboratory for Biomedical Photonics, Collaborative Innovation Center for Biomedical Engineering, School of Engineering Sciences, Huazhong University of Science and Technology, Hubei, China; ^3^School of Mathematics and Economics, Hubei University of Education, Hubei, China

**Keywords:** Lasso-based model, neuronal morphology reconstruction, neuronal image, model optimization, branch points

## Abstract

Reconstruction of neuronal morphology from images involves mainly the extraction of neuronal skeleton points. It is an indispensable step in the quantitative analysis of neurons. Due to the complex morphology of neurons, many widely used tracing methods have difficulties in accurately acquiring skeleton points near branch points or in structures with tortuosity. Here, we propose two models to solve these problems. One is based on an L1-norm minimization model, which can better identify tortuous structure, namely, a local structure with large curvature skeleton points; the other detects an optimized branch point by considering the combination patterns of all neurites that link to this point. We combined these two models to achieve optimized skeleton detection for a neuron. We validate our models in various datasets including MOST and BigNeuron. In addition, we demonstrate that our method can optimize the traced skeletons from large-scale images. These characteristics of our approach indicate that it can reduce manual editing of traced skeletons and help to accelerate the accurate reconstruction of neuronal morphology.

## Introduction

Neuron reconstruction is an important technique in many areas of brain research to identify neuron types, examine neuronal connections, or investigate neuronal circuits. It has therefore been a focus of neuronal image analysis for years (Meijering, 2010; Lu, 2011; Peng et al., 2015). Neuron reconstruction is essentially an extraction of connected skeleton points from a brain imaging dataset (Parekh and Ascoli, 2013; Li et al., 2019). Skeleton points are divided into three classes: branch points, intermediate points, and terminal points. As a series of advances have been made in molecule labeling (Luo et al., 2008; Ugolini, 2010; Jefferis and Livet, 2012) and imaging techniques (Ragan et al., 2012; Silvestri et al., 2012; Gong et al., 2013, 2016; Osten and Margrie, 2013; Liu et al., 2017), nowadays, nearly the complete morphology of a neuron can be visualized at the cellular level in a brain imaging dataset. However, automatic generation of accurate skeleton points faces difficulties, which originate from two typical morphological properties of neurons. One is that the neuronal morphology includes the tortuous structures, in which the curvature of skeleton points is large. In addition, the radii and signal intensity vary a lot for neurites near branch points, which form a complex morphology. These two features are commonly found in neurons but cannot easily be captured by a parameter model.

**Figure 1:**
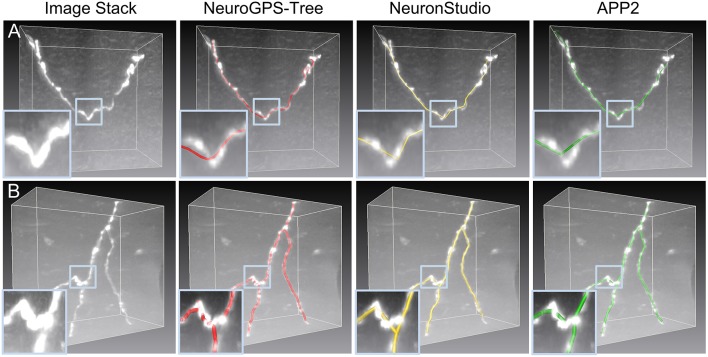
Initial skeletons as determined by tracing algorithms in NeuroGPS-Tree (red), NeuronStudio (yellow), and APP2 (green) require optimization. **(A)** A dataset with a tortuous neurite. The inserts show an enlarged view of the blue squares in **(A)** and illustrate a sharp direction change of the neurite, which induces difficulties in detecting the neurite skeleton; **(B)** The complex branch structure of neurites and the initially calculated corresponding skeletons. Inserts: The enlarged view in **(B)** shows neurite structures and skeletons near the branch point in more detail.

In neuron reconstruction, many methods focus on tracing neurite segments in challenging cases, i.e., fuzzy or broken segments in noisy environments. Some best available methods are listed here, such as graph-based models (Peng et al., 2011; Turetken et al., 2011; Basu et al., 2013; De et al., 2016), principle curve models (Bas and Erdogmus, 2011; Li et al., 2016; Quan et al., 2016), iterative back-tracking (Liu et al., 2018), optimization models (Zhao et al., 2011; Skibbe et al., 2018), minimal path approaches (Lee et al., 2012; Yang et al., 2018), learning structured features (Gu et al., 2017) etc. These methods can automatically extract skeleton points and branching points, which drop into three-dimensional tubular region in neuronal images and construct the geometrical structure of the neuron. Most of these methods behave well in the reconstruction of sparsely distributed neurons. A few of methods involve in identifying individual neurons in the presence of packed neurites (De et al., 2016; Quan et al., 2016). However, for most of these methods, the smoothness of a neurite is an important premise (Skibbe et al., 2018), and the branch point is often handled after acquiring skeletons of neurites (De et al., 2016; Radojević et al., 2016). This indicates that the accurate localization of the positions of intermediate points and branch points in tortuous neurites has barely been explored. In the reconstruction of neurons including tortuous structures, some commonly used methods (Rodriguez et al., 2009; Xiao and Peng, 2013; Quan et al., 2016) fail to determine the accurate positions of the skeleton points in some neuronal structures (Figures 1A,B). This decreases the accuracy of a morphological analysis and leads to difficulties in making conclusions.

A few methods have been proposed for correcting the reconstructed skeleton to better reflect real morphology. For example, Tsai et al. presented an effective model-based method for identifying optimized branch points (Tsai et al., 2004). In this study, the skeleton of a neurite is approached as a line in the least-squares sense and the branch point is refined iteratively as a point that is nearest to these lines. Vasilkoski et al. (Vasilkoski and Stepanyants, 2009) built a method for automatically revising the position of skeleton points including branch and intermediate points. This method is based on the premise that the skeleton is smooth and that the optimal path will, therefore, pass through the high-intensity region in the image. In addition, wavelet transformation (Dima et al., 2002) was used for detecting the optimized branch points. De et al. proposed a two-step tracing approach to address the segment crossovers based on the digraph matrix-forest theorem, in which identifying the accurate position of a branch point is an important issue (De et al., 2016). Radojevi'c et al. developed a fuzzy logic-based system to detect branch points in 2-dimensional images (Radojević et al., 2016). Radojevi'c and Meijering further adopted probability hypothesis filtering to tackle a similar problem in 3-dimensional images (Radojević and Meijering, 2017). In summary, these methods are reasonable and effectively optimize morphologies in many cases. However, the issue of fully considering the presence of tortuous neurite segments and the complex structures around branch points persists.

In fact, it is hard to define whether a skeleton is the optimal one to reflect the real tubular structure, due to lack of standard definition to describe this optimal skeleton. However, it is feasible for us to improve the traced skeletons based on comprehensive consideration of morphological features in tubular structures, such as its tortuosity, signal intensity, and radii changes around local branch point. Based on this information, we presented two optimization models to identify intermediate points and branch points from the initial skeletons. For the first model, we analyzed the morphological characteristics of neurites and found that tortuous elements are sparsely distributed. Here, the sparsity indicates that no or few skeleton points in a segment have a large curvature. Inspired by the Lasso method (Tibshirani, 1996; Tibshirani et al., 2005), we constructed an L1-norm minimization model (Candes et al., 2008) for detection of optimized intermediate points. For the second model, we considered that the real structure of neurites around branch points can be decomposed into three skeleton pieces. Any two of these decomposed elements can be combined into a new skeleton and all skeletons should contain the branch point. This basic fact inspired our procedure for detection of optimized branch points from the initial skeleton. Furthermore, taking the initial reconstructed skeleton of a neuron as input, we integrated these two models and ran them on the original images. An optimized skeleton for this single neuron can be achieved. In our model, the optimized skeleton is a solution to the optimization models and the points are shifted to regions with local maximums, which are generally considered as real skeleton points. This mechanism ensures that the optimized skeleton is closer to real structure, rather than initial skeleton. We demonstrate the performance of the presented method by identifying skeletons of neurons with tortuous structures and complex branch-structures. We also applied our models to initial skeletons from public datasets with noisy backgrounds, i.e., the BigNeuron project (Peng et al., 2015). Furthermore, we demonstrated that our method unifies initial skeletons derived by different algorithms applied to the same dataset, thereby reducing the variability in the reconstruction provided by different algorithms. This may prove very helpful for the morphological analysis of neurons.

## Materials and Methods

### Detection of the Optimized Skeleton of Reconstructed Neurons

The neuronal skeleton generated by reconstruction algorithms may deviate from real neuronal neurites in images, due to the presence of tortuous neuritis, and the complexity of the branch structure. These factors will reduce the accuracy of neuronal morphology quantifications. In this case, we developed two methods, based on an L1-norm minimization model (Tibshirani et al., 2005; Candes et al., 2008), for optimized neurite skeleton generation, and branch point detection. Descriptions of the two methods are presented in the following two sections.

### Detection of the Optimized Neurite Skeleton

The reconstructed skeleton of a neurite is composed of a series of sequential points identified by the tracing algorithm (Rodriguez et al., 2009; Peng et al., 2015; Quan et al., 2016). To detect the optimized neurite skeleton, it is necessary to accurately identify tortuous structures. Here, our solution is based on the following premises: tortuous neurite structures are sparsely distributed and most of the neurite morphologies remain smooth, analogous to a signal sequence of zero and non-zero values representing the smooth and tortuous parts. This is a common situation that can be handled by a Lasso-based model (Tibshirani, 1996). In addition, we also considered that a skeleton point should maintain the maximum image intensity in its neighborhood region. Based on these considerations, we designed our Lasso-based detection model of the optimized skeleton, given by

(1) $$\begin{array}{l} \left(p_{2}^{*},p_{3}^{*},\cdots \;,p_{n-1}^{*}\right)\\ =\underset{p_{2},p_{3},\ldots ,p_{n-1}}{\arg\min}\left(\overset{n-1}{\underset{i=2}{\sum }}g\left(p_{i}\right)+\overset{n-1}{\underset{i=2}{\sum }}\lambda {∥2p_{i}-p_{i-1}-p_{i+1}∥}_{L_{1}}\right) \end{array}$$

Here, solving the lasso-based model (Equation 1) is an iterative process. In this optimization process, we fixed terminal points *p*_1_ and *p*_*n*_ and updated point *p*_*i*_ between the terminal points iteratively. The optimized skeleton of the neurite is represented by a series of points *p*_1_, *p*${}_{2}^{*}$, *p*${}_{3}^{*}$, …, *p*${}_{n-1}^{*}$, *p*_*n*_. The first term in Equation (1) implements the search of local intensity maxima for a skeleton point *p*_*i*_ (Fashing and Tomasi, 2005). In the second term, ∥2*p*_*i*_-*p*_*i*−1_-*p*_*i*+1_∥ is closely related to the curvature of the point *p*_*i*_. Large value of ∥2*p*_*i*_-*p*_*i*−1_-*p*_*i*+1_∥ indicates that the point *p*_*i*_ is included in a local tortuous structure of a neurite. In contrast, a small value corresponds to *p*_*i*_ being in a smooth structure. The second term implements the sparsity of tortuous structure, namely, when minimize equation (1), no or few skeleton points have a large curvature and most of them approach zeros. The parameter λ in the second term controls the sparsity of the tortuous structure and high λ value indicating a strong sparsity. ∥ ∥_L1_ is the L1-norm. The function *g*(*p*_*i*_) in this optimization problem (1) (Equation 1) is given by

(2) $$\begin{array}{r} g\left(p_{i}\right)=-\sum_{p\in \Lambda_{i}}s\left(p\right)\exp\left(-\frac{∥p-p_{i}|{|}_{{}_{2}}^{2}}{2\sigma^{2}}\right) \end{array}$$

Here, the index Λ_*i*_is the neighborhood region of the point *p*_*i*_, where *p*_*i*_ represents the 3-dimensional coordinates of this point. When the coordinate elements of *p*_*i*_ are integers, *p*_*i*_ is regarded as a voxel. *p* has the same definition as *p*_*i*_. *s*(*p*) is the intensity at the voxel *p*, ∥ ∥_2_ represents the L2-norm, and σ is a predetermined scalar.

We further modified the Equation (1) into a constrained optimization problem given by

(3) $$\begin{array}{r} \begin{array}{c} \underset{p_{2},p_{3,\cdots \;,}p_{n-1}}{\min}\;\overset{\text{n-}1}{\underset{\text{i=}2}{\sum }}\lambda ∥d_{\text{i}}{∥}_{L1}+\overset{n-1}{\underset{i=2}{\sum }}g\left(p_{i}\right)\\ \text{subject to }d_{i}=2p_{i}-p_{i-1}-p_{i+1}\; \end{array} \end{array}$$

The Equation (3) can then be converted into the following optimization problem with the augmented Lagrangian method (Hestenes, 1969; Rockafellar, 1973).

(4) $$\begin{array}{l} \underset{r,d,p}{\min}L\left(\text{r,d,p}\right)=\overset{\text{n -}1}{\underset{\text{i =}2}{\sum }}\lambda ∥d_{\text{i}}{∥}_{L1}+\overset{n-1}{\underset{i=2}{\sum }}g\left(p_{i}\right)\\ \;+\overset{\text{n -}1}{\underset{\text{i =}2}{\sum }}<r_{\text{i}},2p_{i}-p_{i-1}-p_{i+1}\text{-}{\text{d}}_{\text{i}}>\\ \;+\frac{1}{2\mu }\overset{n-1}{\underset{i=2}{\sum }}∥d_{i}-\left(2p_{i}-p_{i-1}-p_{i+1}\right)|{|}_{2}^{2} \end{array}$$

The solution of Equation (4) can be effectively obtained by the split Bregman algorithm (Goldstein and Osher, 2009; Ye and Xie, 2011). The pseudo code that solves Equation (4) is shown in Table 1. This algorithm solves Equation (4) as follows:

**Table 1:** Pseudocode for solving Equation (4).

*While (terminal condition is not satisfied) do* $p^{k+1}=\arg\min L\left(r^{k}\text{,}{\text{d}}^{k}\text{, p}\right)=\overset{n-1}{\underset{i=2}{\sum }}g\left(p_{i}\right)+\overset{\text{n -}1}{\underset{\text{i =}2}{\sum }}<{r_{i}}^{k},2p_{i}-p_{i-1}-p_{i+1}\overset{k}{\underset{i}{\text{- d}}}>$
$\begin{array}{c} d^{k+1}=\arg\min L\left(r^{k}\text{,}\text{d}\text{,}{\text{p}}^{k+1}\right)=\overset{n-1}{\underset{i=2}{\sum }}\lambda ∥d_{i}{∥}_{L1}+\overset{\text{n -}1}{\underset{\text{i =}2}{\sum }}<{r_{i}}^{k},2\overset{k+1}{\underset{i}{p}}-\overset{k+1}{\underset{i-1}{p}}\\ -p_{i+1}^{k+1}{\text{- d}}_{i}>\\ +\frac{1}{2\mu }\overset{n-1}{\underset{i=2}{\sum }}∥d_{i}-\left(2p_{i}^{k+1}-p_{i-1}^{k+1}-p_{i+1}^{k+1}\right)|{|}_{2}^{2} \end{array}$
${r_{i}}^{k+1}={r_{i}}^{k}+\left(2p_{i}^{k+1}-p_{i-1}^{k+1}-p_{i+1}^{k+1}\right){\text{- d}}_{i}^{k+1}\left(i=2,\cdots \;,n-1\right)$
*end while*

The above procedure was carried out five times to achieve the optimal solution. The skeletons reconstructed by the tracing algorithm are used as initial points for solving Equation (4). Note that the number of iterations is an experimental parameter. In our analysis, an increase in the number of iterations did not improve the identification of the optimized skeleton (data not shown).

### Detection of the Optimized Branch Point

The neuronal morphology can be modeled as a tree structure. When a neurite connects to another, it makes a contact with one of its terminal ends, which can be regarded as the branch point. Many tracing algorithms have difficulties in detecting the optimized branch point when the physical branch point is surrounded by tortuous neurite structures or when sudden changes in neurite diameter and signal intensity occur (Figure 1B). We observed that in the proximity of the branch point, the sparsity of tortuous structures can still be kept, namely, no or few skeleton point of a neurite have a large curvature. In this sense, our model (Equation 1) can effectively identify this structure. In addition, the neurite branch structure is often formed by three segments. Any two segments can form a new neurite skeleton in which the branch point is included. This means that during the branch point identification, we should consider all neuronal segments that link to the branch point. To identify a branch point, we first used, as described in the previous section, a Lasso-based model to produce the optimized skeleton of the combined neurite segments. Then we designed a model to optimize the position of the current branch points. The specific procedure was as follows:

Before detecting the optimized branch point in a reconstructed neuron, we searched for a branch point and extracted its related local skeletons, represented by *S*_1_ = (*p*_11_, *p*_12_,…, *p*_1*s*_,…, *p*_1*n*_) and *S*_2_ = (*p*_21_, *p*_22_, …, *p*_2*m*_), where *p*_1*s*_is equal to *p*_21_ as the branch point. The skeleton *S*_1_ contains the branch point and can be decomposed into two segments *S*_11_ = (*p*_11_, *p*_12_,…, *p*_1*s*_) and *S*_12_ = (*p*_1*s*_,…, *p*_1*n*_). To reduce the computational costs, *m* and *n* were set to 21 and 16, respectively. These empirical values were used for extracting a local branch structure that contains the target branch point and its adjacent neurites. The values selection is based on a rule that the extracted information is enough to optimize this branch point. In this sense, large values indicate that unnecessary neurite information is included and arises the computation time. Small values lead to insufficient information that reduces the optimization accuracy. We chose these values based on the real size of branch structures in general and they are fixed in our algorithm.

The steps for detecting a branch point were the following:

*Step* (1) Adjust the skeleton *S*_1_ with Equation (4), denoted by *S*_1_^*^. Perform a sample operation on *S*_1_^*^ to assure that the Euclidean distance between any two neighboring resampled points equals 1 μm.
*Step* (2) Convolute the resampled skeleton with a 3D Gaussian template (sigma = 1.73) and generate an image in which the signal intensities of all skeleton points are almost the same and bigger than those of non-skeleton points.
*Step* (3) Amend the branch point *p*_21_ according to the model Equation (5) in which the information from the convolution image in *Step* (2) and the skeleton *S*_2_ are used.
*Step* (4) Use Equation (4) to adjust the *S*_2_ skeleton points *p*_22_*, p*_23_*,…,p*_2, *m*−1_accordingly, with fixed terminal points *p*_21_ and *p*_2m_.
*Step* (5) Repeat *Steps* (3) and (4) until the position of *p*_21_converges, which is denoted as *p*${}_{21}^{*}$.
*Step* (6) Recombine skeletons *S*_12_and *S*_2_into a single neurite skeleton and use *S*_11_ as the other neurite. Repeat *Steps* (1)–(5) with these values, generating the branch point *p*${}_{1s}^{**}$.
*Step* (7) Repeat *Step* (6) to generate the branch point *p*${}_{1s}^{***}$ for the recombined skeletons *S*_11_and *S*_2_ with *S*_12_ as the additional neurite.

The average position of the three determined points *p*${}_{21}^{*}$, *p*${}_{1s}^{**}$, and *p*${}_{1s}^{***}$ is regarded as the optimized branch point. In *Step* (2), we generated the synthetic data for detecting the optimized branch point. In this dataset, the signal intensities of all the skeleton points have the maximum value. This ensures that the branch point can be located in the skeleton with a high probability. By contrast, in real images, branch points sometimes have lower signal intensities than other skeleton points, which lowers their chance to be identified in an experimental dataset. In addition, in the synthetic datasets, the signal intensities along the skeleton are almost the same. This ensures that any position in the skeleton can potentially be identified as a branch point with the same chance when other factors are not considered. Therefore, we used a synthetic dataset in *Step* (2) of the procedure for detecting branch points. Note that, we selected the Gaussian kernel width (sigma = 1.73) based on the fact that this kernel width should match with the radius of a neurite, namely, < 2.5 μm (De Paola et al., 2006; Loopuijt et al., 2007) in general. In addition, the optimization position changes slightly in a wide range of sigma value (Figure S1).

In *Step* (3), we improved the branch point positioning of the optimization model according to

(5) $$\begin{array}{l} \underset{p_{bifur}}{\min}g\left(p_{bifur}\right)+\lambda {\left(p_{fx}-p_{bifur}\right)}^{T}\left(dv_{2}d{v_{2}}^{T}+dv_{3}d{v_{3}}^{T}\right)\\ \left(p_{fx}-p_{bifur}\right) \end{array}$$

Here, g (*p*_*bifur*_) is calculated based on Equation (2) in the simulation image that was generated in *Step* (2). The point *p*_fx_ denotes a reference point in the skeleton *S*_2_. This point meets the condition that the distance between *p*_fx_ and any point in the skeleton *S*_1_ is more than three times the voxel size. The fixed orientation vector ***dv***_**1**_forms an orthonormal coordinate system with ***dv***_**2**_ and ***dv***_**3**_. ***dv***_**1**_ is calculated as

(6) $$\begin{array}{r} dv_{1}=\frac{p_{v1}-p_{fx}}{∥p_{v1}-p_{fx}∥} \end{array}$$

*p*_*v*1_denotes a point in the skeleton *S*_2_ and satisfies the condition that the distance between this point and *p*_*fx*_ is more than three times the voxel size.

Note that minimizing the first term in Equation (5) ensures that the branch point is located at the skeleton *S*_1_, while minimizing the second term means that the orientation from the branch point *p*_*bifur*_ to *p*_*fx*_ is similar to the orientation *dv*_1_. Additionally, the branch points that need to be optimized in skeletons *S*_11_and *S*_12_ in *Steps* (6, 7) still satisfy the above description and can be obtained using Equation (5).

### Workflow for Optimizing the Reconstructed Skeleton of a Neuron

**Figure 2:**
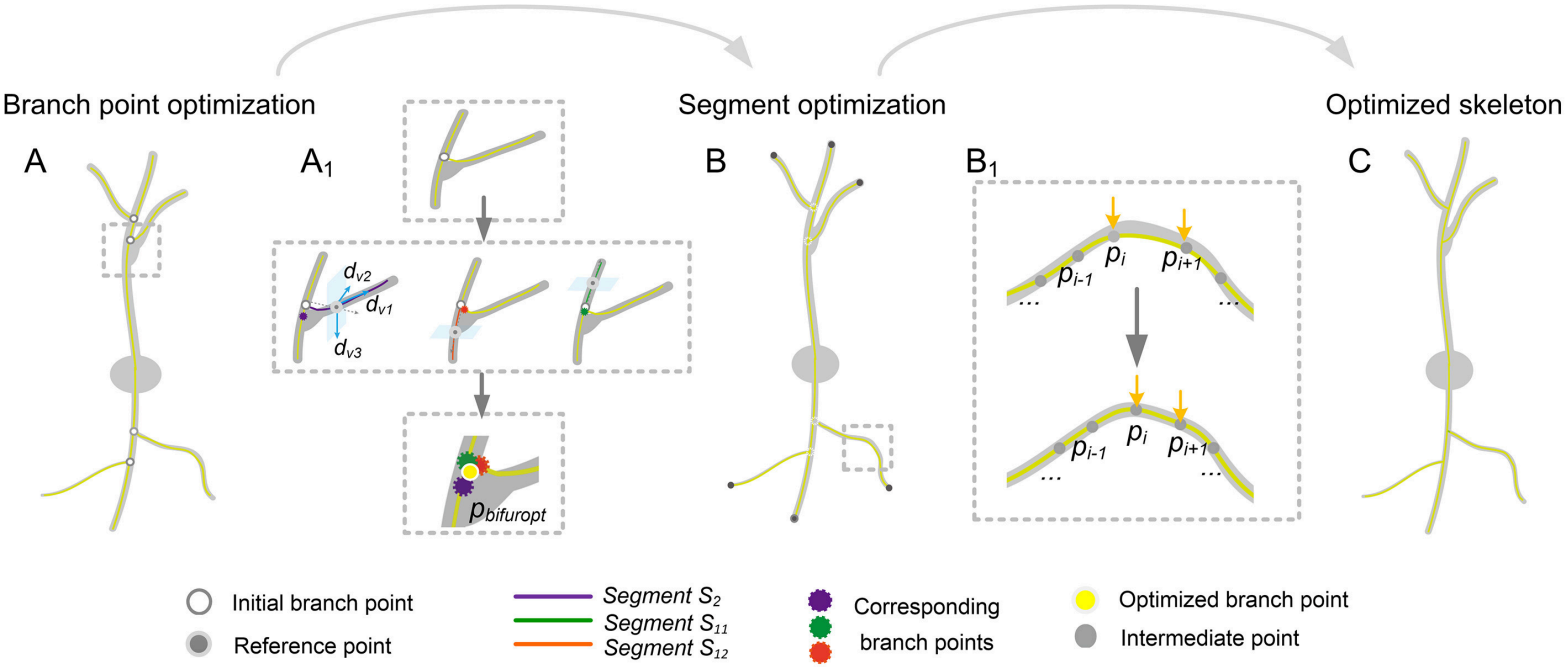
Workflow for identification of the optimized neuronal skeleton. **(A)** Optimizing all branch points in the neuronal skeleton. Detecting the initial branch points of the skeleton and an area (dashed square) that includes an initial branch point (white dot) is enlarged in **(A**_**1**_**)**; **(A**_**1**_**)** The procedure for optimizing branch points. Upper panel: The branch structure (yellow) enlarged from **(A)**. Middle panel: Process of identifying the optimized branch point: The branch structure can be treated as three segments (purple, red, and green) whose one terminal end directly links to the branch point. For one segment (purple), build an orthonormal coordinate system based on its direction. With the help of this coordinate system and a reference point (gray dot with white circle), the optimization model Equation (5) is constructed, and the initial branch point is modified (purple dot, left image). For the other two segments (red and green), a similar model is constructed based on a corresponding orthonormal coordinate system, and the corresponding branch points are generated (red and green marks in the middle and right image, respectively). Bottom panel: the optimized branch point (yellow mark) is determined by averaging the positions of the three calculated branch points (purple, green, and red marks); **(B)** Optimizing the segments which are composed of intermediate points with fixed branch and terminal points; **(B**_**1**_**)** Optimization of intermediate points. An enlarged view of a tortuous neurite in **(B)** is shown. In this area, some intermediate points (*p*_*i*_and *p*_*i*+1_, yellow arrows) deviate from their real positions (upper image). After optimization of the skeleton, the positions of these points are adjusted (lower image); **(C)** Optimized skeleton of this neuronal tree.

The reconstructed skeleton of a neuron can be represented by a tree structure. In this structure, each skeleton has two ends. One end has no links. The other end directly links to another skeleton and is defined as the branch point. Depending on this tree structure, all branch points in a reconstructed skeleton can be successfully detected. Based on the detected branch points and the terminal points, the initial skeleton segment which connects to them can be detected. Thus, the methods described in the previous sections can be applied to identify the optimized branch points and segments and finally determine an optimized skeleton of a neuron. The workflow is as follows (see Figure 2):

*Step* (1) Detect all branch points in the reconstructed tree.
*Step* (2) Amend the branch points as described in the previous subsection.
*Step* (3) Adjust the corrected branch points and adapt all skeleton segments as described in the first subsection of this method section.

### Measuring Morphological Features of a Neuronal Reconstruction

For measuring the morphological changes before and after optimizing the traced neurites, we selected two indexes, neurites length, and local branching angle, and used software Amira to calculate them (Stalling et al., 2005). When using “SpatialGraphToLineSet” module in this software, the length of every neurite segment can be automatically computed and then the total length can be generated. In the calculation of a branching angle, we used “3D angle” tool in the measuring module. One middle point (branching point) is fixed and two local segments that directly link to the branching point are manually determined. Both of the local segments have the same length (10 μm). After finishing these settings, we can get the branching angle.

### Generation of Synthetic Datasets

We took the following steps to generate a dataset that includes a synthetic neurite with tortuous structure.

**Figure 3:**
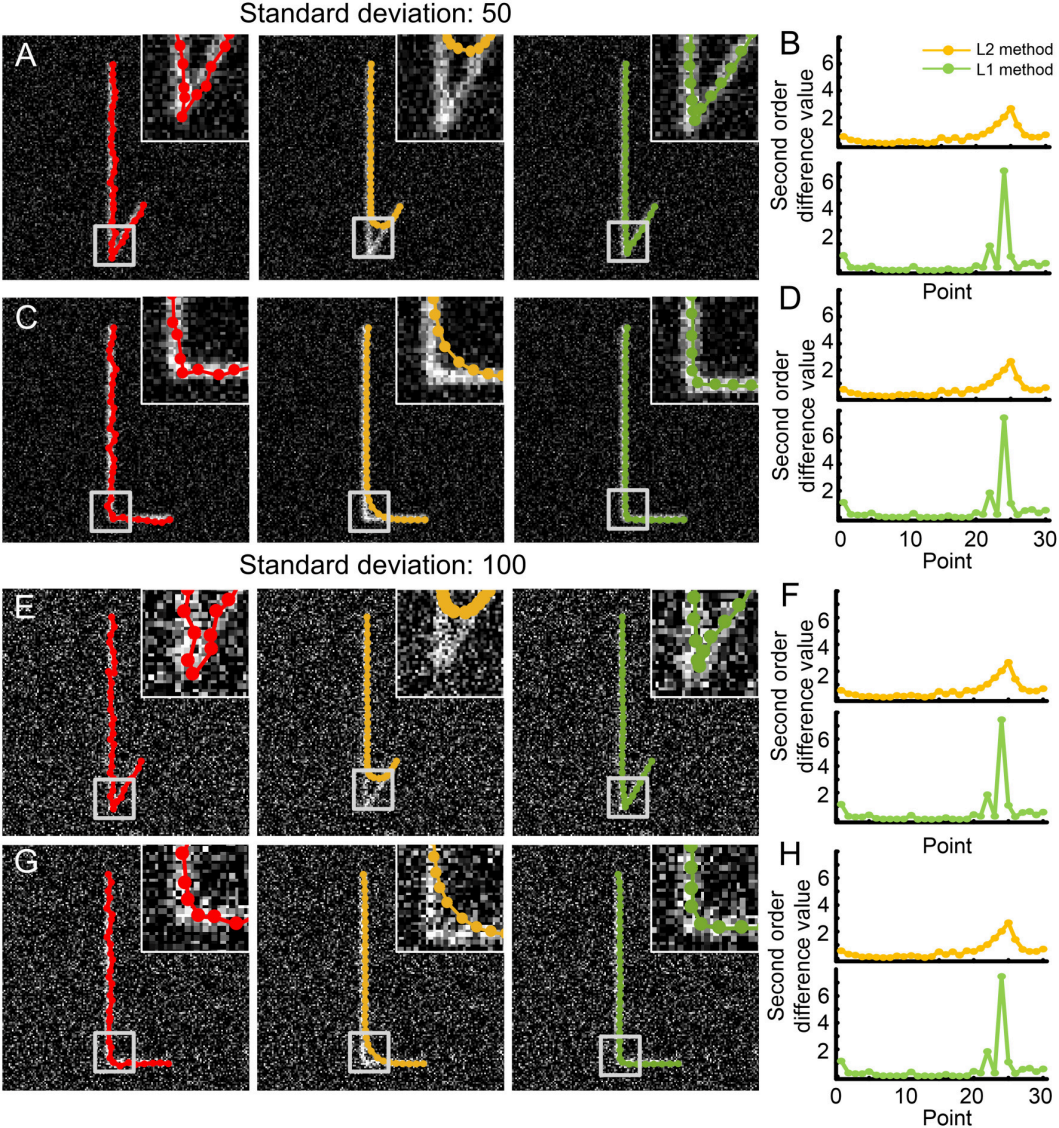
Performance comparison between the L2 minimization-based method and our model to detect the skeleton of tortuous structures in a synthetic dataset. **(A)** A dataset polluted with Gaussian white noise (mean: 0, standard deviation: 50) contains a neurite (initial skeleton, red) with a 30-degree direction change. The skeletons as determined by the L2-minimization and our model are shown in yellow and green in the middle and right panels, respectively. Insert: The tortuous structure of the neurite and the corresponding skeletons; **(B)** Distance measured between two adjacent intermediate points on the detected skeletons from both methods (upper panel: L2 minimization-based method, bottom panel: our model); **(C)** Initial and detected skeletons acquired using the same methods as in **(A)** in a dataset with a weaker 90° tortuosity; **(D)** Distance of adjacent skeleton points in **(C)** as described in **(B)**; **(E)** Dataset polluted with Gaussian white noise of a higher noise level (standard deviation: 100). The neurites have the same structure as in **(A)**, and their initial and detected skeletons are generated in the same way; **(F)** Distance of skeleton points in **(E)** measured according to **(B)**; **(G)** The dataset with 90-degree tortuosity as shown in (**C**) but with the same higher noise level applied to **(E)**; **(H)** Distance of adjacent skeleton points in **(G)** calculated as in **(B)**.

(1) Generate a binary image: We first generate a three-dimensional image stack with predetermined sizes. The values of voxels in this image are set to zero. Then, we select the locations of two terminal points and a specific intermediate point. We use a fold line to connect these three points and ensure the curvature of the selected intermediate point is nonzero. Finally, we find the voxels that along the fold line and set their intensity values to one.

(2) Generate a synthetic dataset: We use a three-dimensional Gaussian kernel (kernel width = 1.73) to convolute the binary image, normalized the convoluted images (maximum value = 255) and add Gaussian white noise into it. The above process is originated from the model that describes how to collect the light microscopic images (Agard et al., 1989). Note that, the curvature of the specific intermediate point has nonzero value and corresponds to the tortuous structure. By changing the curvature value of this point, we can generate synthetic neurites in different tortuous level. We further used these synthetic datasets to illustrate the concept and effectiveness of the proposed optimization model, Equation (1) (Figure 3).

## Results

We evaluated the performance of our model in detecting the skeleton of tortuous structures. Synthetic datasets were generated using the method introduced in section 2.6. We generated four image stacks that had the same size of 156 × 156 × 57 voxels but with different noise level. The noise in the images is controlled by Gaussian white noise (mean value: 0, standard deviation: 50 or 100). The tortuous level in these image stacks varies according to the curvature change of the specific intermediate point. As the angle variation is closely related to curvature. Here, we used an angle that associated with the specific intermediate point to quantify the curvature changes. The angle is achieved between two segments, which are formed with the previous and consecutive skeleton point of this specific intermediate point. In the synthetic datasets, 30 and 90 degrees were set. The initial skeleton was generated by adding random perturbation on the predetermined fold line in synthetic datasets (Section Generation of synthetic datasets). We optimized these initial skeletons and used the optimized results for evaluating the model.

For an image stack with low-level noise and a 30-degree direction change (Figure 3A), our model successfully identified the skeleton of the tortuous structure (Figure 3A, right panel, green) from the initial skeleton (Figure 3A, left panel, red). In contrast, the L2 minimization-based method (Vasilkoski and Stepanyants, 2009) failed under these conditions (Figure 3A, middle panel, yellow). In fact, the L2 minimization-based method contains a term that describes the sums of square deviations of adjacent skeleton points. This term helps to keep the smoothness of the skeleton, with all second-order differences between adjacent skeleton points being infinitely close to zero. The L1 minimization-based method only requires the above situation for most values. So, when a tortuous structure is detected, the corresponding second-order differences diverge from zero. The L1 minimization-based method can capture these non-zeros values due to their sparse property, while the L2 minimization-based method fails (Figure 3B). This may be an explanation why our L1 minimization-based method can identify the skeleton of a tortuous structure. When we decreased the tortuosity of the structure to a 90-degree direction change, similar results were achieved (Figures 3C,D). Furthermore, the L1 minimization-based method established the skeleton of structures with different tortuosity levels well, even in a high-noise environment (Figures 3E–H). From these results, we concluded that our model can detect the optimized skeleton of neurites with tortuous structure.

**Figure 4:**
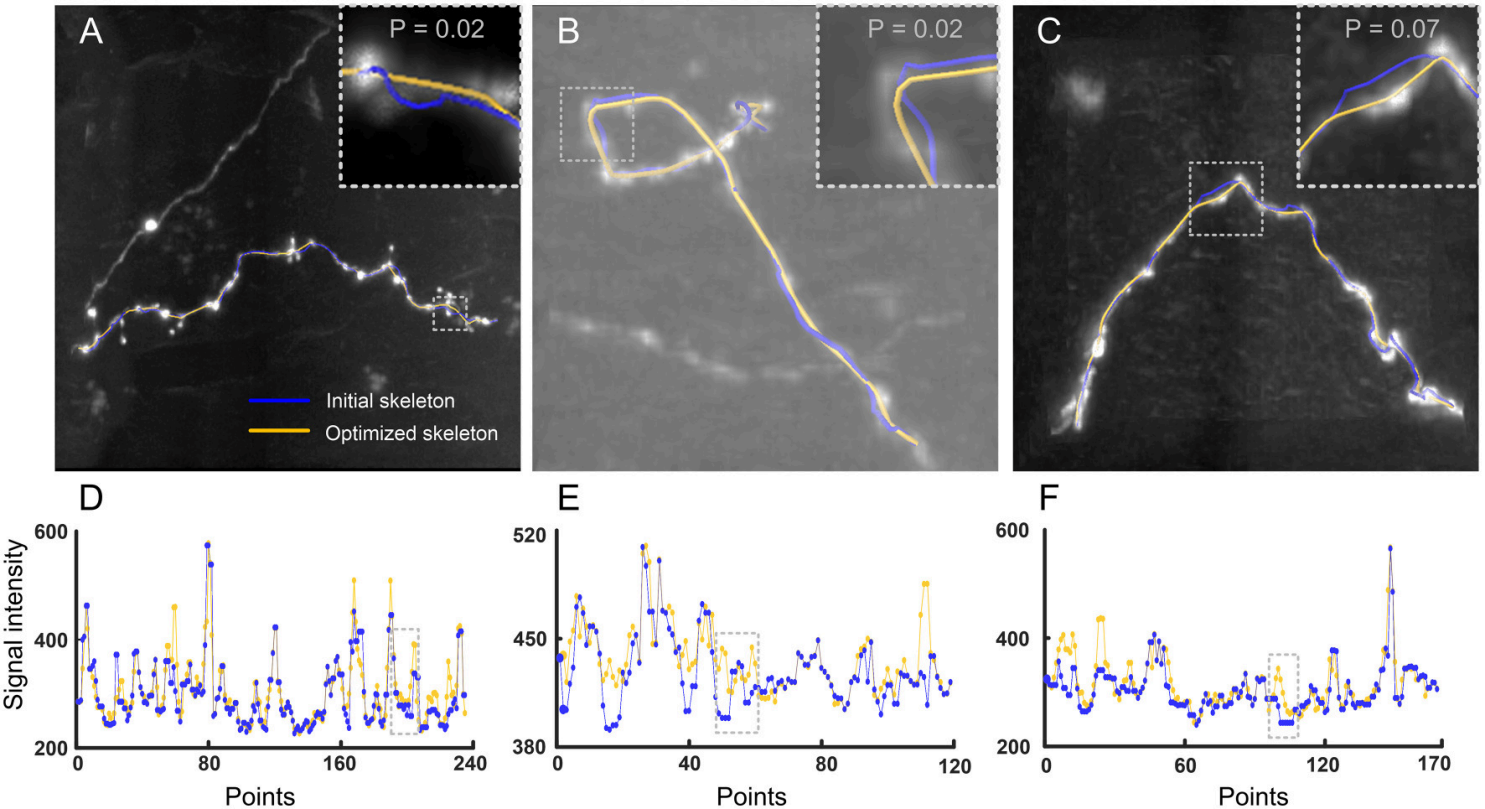
Performance of our model in optimizing skeletons in experimental datasets. **(A)** A dataset with a large signal intensity range. The initial and optimized skeletons are presented in blue and yellow, respectively. Insert: Higher magnification of the dashed square. *p*-value: Kolmogorov-Smirnov test on the initial and optimized skeleton intensity values, see dashed rectangle in **(D)**; **(B,C)** Dataset with tortuous structures in a picture with high and low background noise, respectively. All other descriptions are similar to **(A)**; **(D)** Signal intensities of every intermediate point along the initial (blue) and optimized (yellow) skeleton as shown in **(A)**. The dashed window corresponds to the insert in **(A)**; **(E,F)** Signal intensity variations of the skeletons shown in **(B,C)**, all other details as in **(D)**.

We also evaluated the performance of our model in identifying skeletons in experimental datasets. These datasets included three image stacks with a size of 101 × 101 × 51 voxels. One dataset has a large dynamic range of signal intensities (Figure 4A) and the other two present tortuous neurites (Figures 4B,C). For these datasets, the initial (blue) and optimized (yellow) skeletons are shown in Figures 4A–C. Here, the optimized skeleton refers to the skeleton generated by solving Equation (4). The initial skeleton was provided by NeuroGPS-Tree (Quan et al., 2016). Our results indicate that compared to the initial skeleton, the skeletons determined by our model are closer to the real skeleton of the neurite. We further illustrated this point by quantitative analysis. It is well acknowledged that each point of the real skeleton has the local maximum image intensity (Wang et al., 2011; Quan et al., 2016). Based on this, we compared the image intensities along the initial and the optimized skeleton. Specifically, for each optimized skeleton point, we searched a point in the initial skeleton that was nearest to this optimized skeleton point. Our results suggest that a certain number of optimized points have higher signal intensity than those of the initial skeleton points (Figures 4D–F). We also determined statistically significant differences between the signal intensity values of optimized and initial points using the Kolmogorov-Smirnov test, on the intensity values shown in the dashed rectangles of Figures 4D–F. The size of the rectangle is dependent on the tortuous structure that needs to be covered. The corresponding images are shown in the inserts of Figures 4A–C in which neurites exhibit a high dynamical range and tortuous structures. The statistical results demonstrate a significant difference between the corrected skeleton and the initially calculated points (*p*-values of 0.02, 0.02, and 0.07). From these test results, we concluded that our model can generate a corrected skeleton that better reflects reality than the initial skeleton.

**Figure 5:**
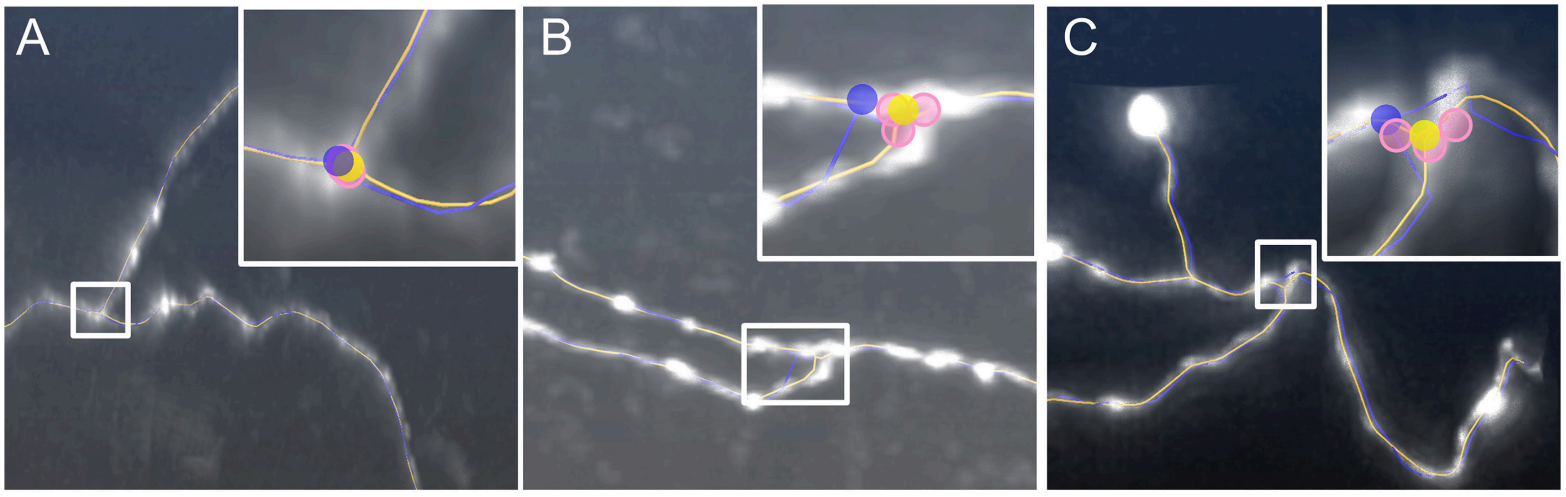
Identification of optimized branch points by our model. The signal intensity changes in neurites around the branch point in **(A)**, the diameter varies along neurite segments and the segments form a small angle in **(B)**, and the structure near the branch point is tortuous in **(C)**. Inserts: Higher magnifications of the white squares. The initial position (blue dots) deviates from the branch point location. The optimized branch points (yellow dots) are the average from three corresponding branch points (pink dots). The distances between initial and optimized branch points in **(A–C)** are 1.4, 4.8, and 3.6 μm, respectively.

Next, we examined the performance of our model to identify branch points. We used three image stacks (Gong et al., 2013) for testing our model. The first dataset illustrated a branch structure with smooth neurites, in which the initial branch point (blue dot in Figure 5A) is slightly shifted from the real position. In the last two datasets, the neurites near the branch point exhibit a shape change in terms of signal intensity and segment diameter (Figures 5B,C), and the tortuous structure in Figure 5B appears more challenging. These image characteristics may challenge the current tracing algorithms and the branch points in the initial skeletons (blue dots, inserts of Figures 5B,C) deviate from their real positions. As described in the methods section, our model generated three corresponding branch points (pink dots, inserts of Figures 5A–C) on different joint segments, thereby suppressing interferences induced by the characteristics described above. The average of these three positions determines the optimized branch points (yellow dots, inserts of Figures 5A–C), which can better reflect the real branch point positions. These results indicate that our model is effective for detecting the optimized branch point even under complex conditions.

**Figure 6:**
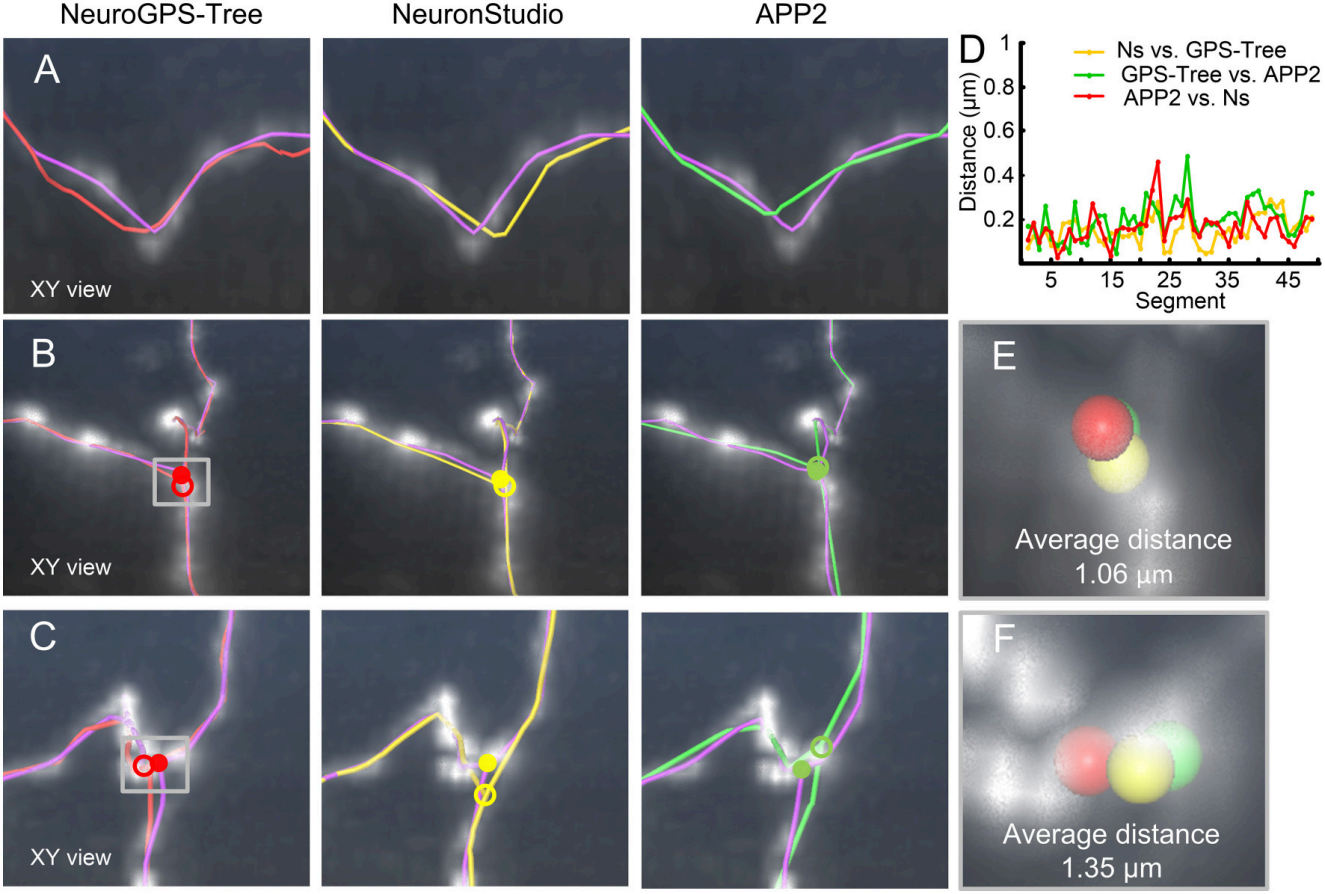
Adjusting skeletons of initial reconstructions derived from NeuroGPS-Tree, NeuronStudio, and APP2 using our model. **(A)** A neurite with a tortuous structure (gray square). The initial skeletons (red, yellow, and green, NeuroGPS-Tree, NeuronStudio, and APP2, respectively) and the optimized results (purple, our model) are shown; **(B,C)** Datasets with a branch structure (gray square), **(C)** contains additionally tortuous neurites near the branch point. The branch point position is shown before (hollow dots) and after (solid dots) the optimization by our model; **(D)** Distance between optimized skeletons generated from different initial reconstructions of the tortuous structure in **(A)**. Ns: NeuronStudio, GPS-Tree: NeuroGPS-Tree; **(E,F)** Higher magnifications of the branch points in **(B,C)**. Different colored spheres indicate the branch point positions determined by our model, derived from each initial skeleton of other tracing algorithms.

We furthermore verified that our model can effectively correct the skeletons generated by different tracing algorithms. We used NeuroGPS-Tree (Quan et al., 2016), NeuronStudio (Rodriguez et al., 2009), and APP2 (Xiao and Peng, 2013) to evaluate three image stacks. The first image stack contains a tortuous neurite (Figure 6A), and the other two contain a branch structure (Figures 6B,C). The initially traced skeletons are displayed in red, yellow, and green curves, and the initial branch points are represented by hollow dots. These initial skeletons and branch points are different, deviate from reality and were optimized by our model. The optimization results are displayed with xy perspective view (Figures 6A–C), and with xz and yz perspective views (Figure S2). From the optimization results, we conclude that the initially traced results can be corrected to almost the same skeletons (purple curves) and branch points (solid dots).We quantified these properties (Figures 6D–F) by first evaluating the consistency of the corrected skeletons with tortuous structures. We resampled the points on the corrected skeletons from the start point with the same *x*-coordinates to ensure that the distance between the *x*-coordinates of two adjacent points is 1 μm. We took 51 resampled points on each of these three skeleton points. As two adjacent resampled points form a segment, we then separately calculated the distance between the matched-pair segments (Lee et al., 2007). A segment on the skeleton and its nearest segment on the other skeleton were defined as a matched-pair segment. The small distances between these matched-pair segments indicate consistency among the corrected skeletons. The quantified results (Figure 6D) also indicate that these corrected skeleton points have similar locations. Similarly, the average distance between any two optimized branch points (red, yellow, and green solid dots in Figures 6B,C) is presented in Figures 6E,F. The two distance values for Figures 6B,C are 1.06 and 1.35 μm, respectively, vs. 1.74 and 5.23 μm for the initial branch points. These results show that our model can be applied to other tracing methods and creates a consistent skeleton independent of the initially provided parameters.

**Figure 7:**
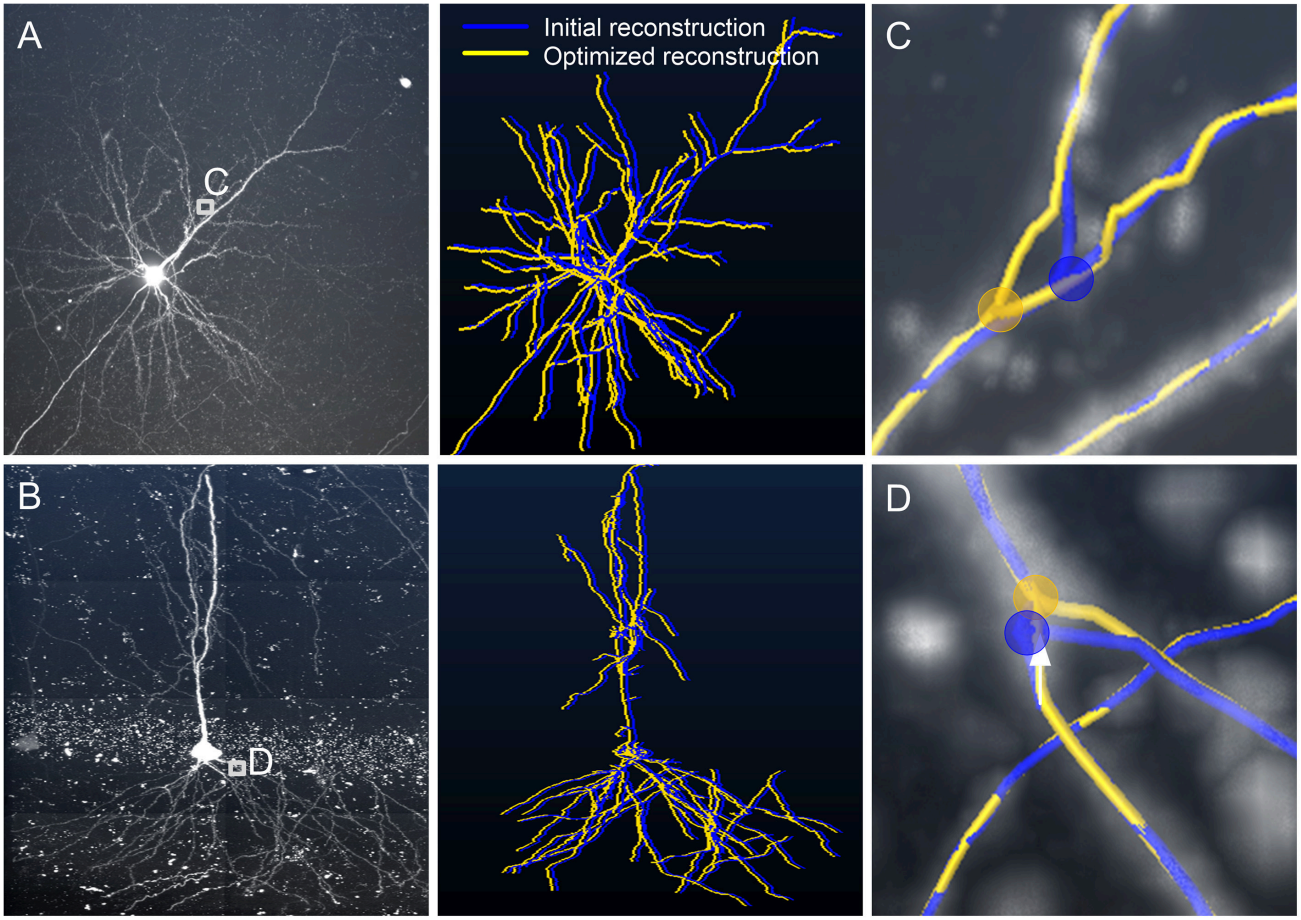
Optimizing neuronal trees from the BigNeuron project with our model. **(A,B)** Left panel: Original data [checked6_mouse_tufts, **(A)**; checked_mouse_korea, **(B)**] from the BigNeuron project. Right panel: Reconstructions before (blue) and after (yellow) optimization. We shifted the optimized reconstruction from the initial one for a clear demonstration; **(C,D)** enlarged views of **(A,B)**, respectively. Location of branch points before (blue dot) and after (yellow dot) optimization. Distances between blue and yellow dots are 3.2 and 2.1 μm.

In addition to the dataset collected with Micro-optical Sectioning Tomography system, i.e., MOST dataset (Gong et al., 2013), our detection method can be applied to other datasets like those from the BigNeuron project. Two typical datasets were selected for this purpose. One dataset includes a pyramidal neuron with an abundance of neurites. Some of these neurites have low signal intensities and are severely masked by noise. The other dataset includes a neuron surrounded by noisy points, especially around the soma area. We reconstructed these two neurons with NeuroGPS-Tree and revised the skeletons with our model. The initial and optimized skeletons seem to be similar (Figures 7A,B, right panels) in most regions. However, the initial tracing failed in a few branch structures with small angles (Figures 7C,D) and the optimized skeleton is superior in locating these branch points. These results indicate that our model can accurately detect skeleton points when a region presents a complex branch structure in a noisy background. We also noted that our model is efficient in terms of computation time. The optimized skeletons for these two neurons in Figures 7A,B were detected in about 202 and 55 s, respectively. The two tests were carried out on a computer workstation (Intel® Xeon^®;^ CPU 3.46 GHz computing platform, Quadro K4000 3G GPU, 192 GB RAM, Windows 7). The data size is 1455 × 1455 × 500 voxels for Figure 7A and 2715 × 4011 × 141 voxels for Figure 7B.

Neurites lengths and branching angles of neurons are important morphological features. We used these two features to measure the traced neurites (Figures 4–7). The detailed operation is described in Section Measuring morphological features of a neuronal reconstruction and their related morphometric measurements can be found in Tables 2, 3. Note that, the datasets in Figure 5A are the same one tested in Figure 6B. Thus, we only listed their measurements in Table 3 to avoid redundant information. The results in Table 2 suggest that our optimization method can yield about 10% change in branching angle and increase more than 200 μm when measuring total length of traced neurites in Figure 7A. In addition, when using NeuroGPS-Tree, NeuronStudio and APP2 to reconstruct the same neurites, the reconstructions in general are different. In this case, our optimization method can make these different reconstructions consistent, as indicated in Table 3. The results show that our optimization method achieves almost the same length (data 1 in Table 3) and local branch angle (data 2–3 in Table 3) from different initial reconstructions, which are consistent with the conclusion drawn from the quantitative results in Figures 6D–F.

**Table 2:** Measurements on initial and optimized reconstructions in Figures 4, 5, and 7.

**Data id (source)**	Total length^*^ (μm)	Total length^**^ (μm)	Local branch angle^*^ (^**°**^)	Local branch angle^**^ (^**°**^)
1 (Figure 4A)	258	252	N/A	N/A
2 (Figure 4B)	138	135	N/A	N/A
3 (Figure 4C)	184	168	N/A	N/A
3 (Figure 5B)	1,035	1,030	49	43
4 (Figure 5C)	276	270	70	71
5 (Figure 7A)	13,611	13,861	41	43
6 (Figure 7B)	18,847	18,818	42	46

* *Initial reconstruction*;

** *Optimized reconstruction; N/A: Not available*.

**Table 3:** Measurements on initial reconstructions from NeuroGPS-Tree (Tree), NeuronStudio (Ns) and APP2, and their optimized reconstructions in Figure 6.

**Data id (source)**	Total length^*^(μm)			Total length^**^ **(μm)**			Local branch angle^*^ **(**^**°**^**)**			Local branch angle^**^ **(**^**°**^**)**		
	**Tree**	**Ns**	**APP2**	**Tree**	**Ns**	**APP2**	**Tree**	**Ns**	**APP2**	**Tree**	**Ns**	**APP2**
1 (Figure 6A)	72	71	66	67	67	68	N/A	N/A
2 (Figure 6B)	276	273	246	270	265	249	56	59	69	63	62	63
3 (Figure 6C)	318	309	311	309	305	309	122	127	96	114	112	108

* *Initial reconstruction*;

** *Optimized reconstruction; N/A: Not available*.

**Figure 8:**
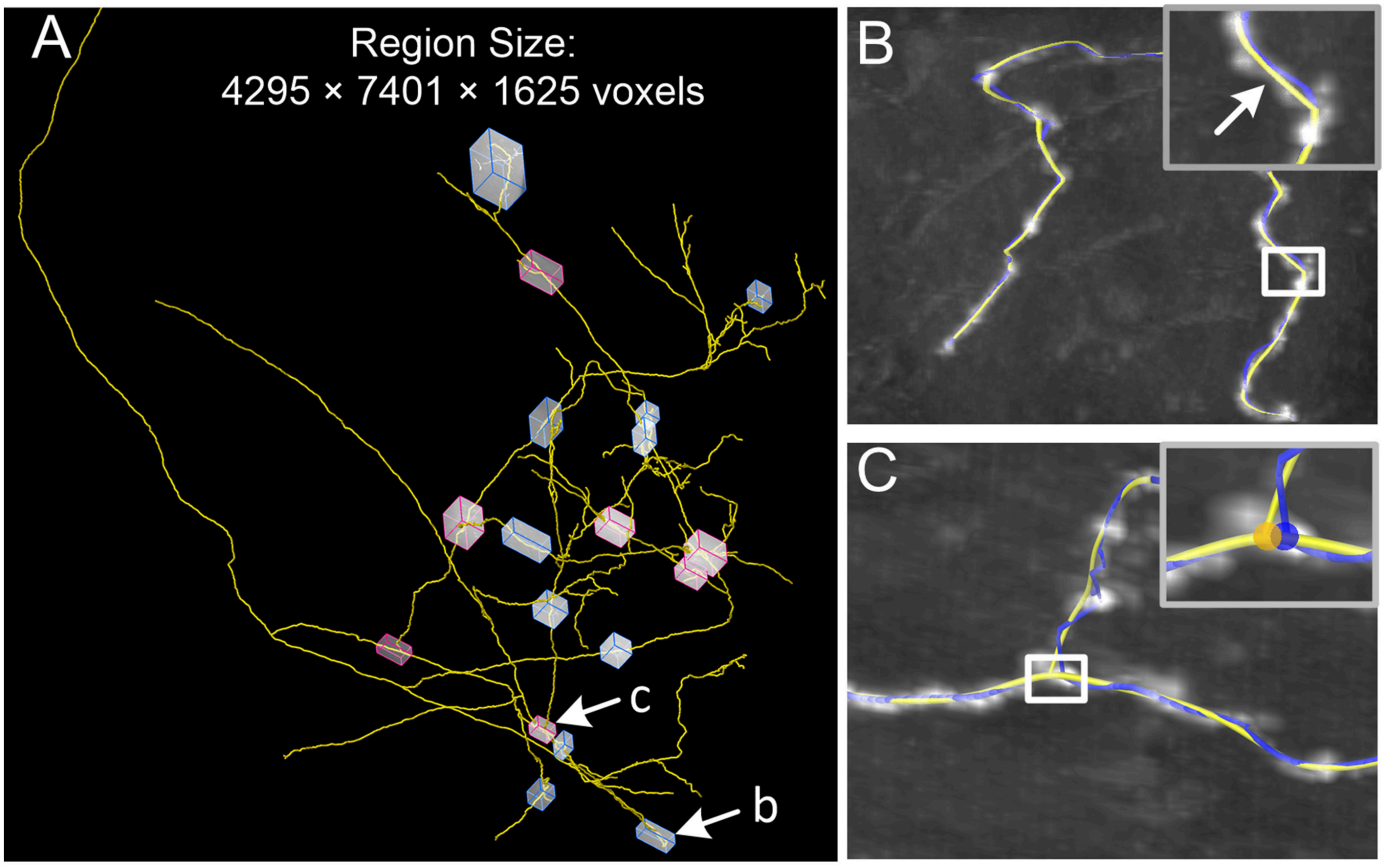
Optimization of a reconstructed skeleton in a large-scale dataset. **(A)** In the initial skeleton (yellow) provided by GTree, 11 locations with tortuous structures (blue blocks) and seven locations with complex branch structures (red blocks) are highlighted. Two typical examples are shown in **(B,C)** as indicated; **(B,C)** A tortuous neurite and a complex branch structure, with the initial (blue) and optimized (yellow) skeletons; Inserts: Higher magnifications of the white squares.

Correcting the initial skeleton in large-scale images is a difficult task. Our identification method has been integrated into our tool, Global Tree reconstruction system — GTree (https://www.biorxiv.org/content/early/2018/01/02/223834), and can correct a large-scale traced skeleton. To verify this point, we used a dataset that contains 4295 × 7401 × 1625 voxels (voxel size, 0.3 × 0.3 × 1 μm^3^; Figure 8A). The original voxels were merged into the new voxels with a size of 0.6 × 0.6 × 1 μm^3^ in our analysis, which helps to weaken the anisotropic property of the dataset. This merged voxel size drops into the range from 0.5 × 0.5 × 0.5 μm^3^ to 2 × 2 × 2 μm^3^, which GTree recommends. There are a certain number of complex structures around branch points and tortuous neurites in this large-scale dataset. We selected 11 locations (blue blocks) where the initial skeleton obviously deviates from the neurite centerlines. Our model can successfully correct these initial skeletons, and a typical example is shown in Figure 8B (white arrow). Additionally, red blocks in Figure 8A indicate areas in which the branch point needs to be adjusted. These initial branch points were revised by our model, and an example is presented in Figure 8C (blue and yellow dots). These results demonstrate that our model can be automatically applied to initial skeletons of axonal datasets, which will help to minimize the manual revision of large-scale skeletons.

## Discussion

At present, many tracing algorithms (Rodriguez et al., 2009; Xiao and Peng, 2013; Quan et al., 2016) have difficulties detecting branch points or intermediate points. This is mainly caused by three issues. The first issue is that a number of methods adapt a simple way to detect branch points around complex branch structures. The detection procedure can be summarized as follows: First, trace a neurite, estimate, and label its shape; second, trace another neurite, and stop tracing when the current tracing point connects to the labeled region or is classified as a background point. Third, after the tracing steps, detect the traced skeleton point that is nearest to the current tracing point, which can be regarded as the branch point. This procedure indicates that an effective detection of a branch point requires the accurate tracing of branch structures and neurite shapes, which is a challenging problem. The second issue causing difficulties for tracing algorithms is that many algorithms (Bas and Erdogmus, 2011; Xiao and Peng, 2013; Li et al., 2016; Quan et al., 2016; Skibbe et al., 2018) neglect the tortuous structure of neurites or even introduce a penalty term to generate a smooth skeleton (Vasilkoski and Stepanyants, 2009). Thirdly, designing a neuron tracing algorithm is a complicated problem. In this case, considering too much situations will increase the algorithm complexity and reduce its robustness. Thus, in the initial skeleton provided by a variety of tracing algorithms, intermediate or branch points commonly deviate from their real positions. A feasible way to overcome these restrictions may as follows. Tracing algorithms provide the initial reconstructions and then these initial reconstructions are corrected automatically. Our method was motivated by the requirement to develop a better approach to automatically optimize the traced neuronal skeletons.

Although the majority of neurites exhibit a smooth structure, tortuous segments exist and are sparsely distributed along neurites. These morphological characteristics prevent existing tracing algorithms from depicting neurite structures correctly, especially in tortuous regions. In essence, a tortuous structure in a mainly smooth neurite can be described as a situation where non-zeros exist in a null sequence. This situation is closely related to the case that a Lasso-based model (Tibshirani et al., 2005) can well handle. Thus, we considered the L1-norm function as a penalty term in our model. Note that employing the L2-norm function in the identification model can accurately describe the smoothness of neurites, but will lead to failures in the identification of the skeleton in tortuous segments (see Figure 3).

Besides the tortuous structures in neurites, the complex branch structure also challenges the existing algorithms for achieving a faithful reconstruction result. Difficulties originate from multiple aspects such as tortuous structures, sharp changes in signal intensity, and different neurite diameters in the proximity of a branch point. Existing parameter models have difficulties in describing these various characteristics and thus fail to detect the branch points in some cases. Instead of constructing a parameter model, we designed an optimization problem for detecting a branch point, which is based on the following facts: A branch point locates where two neurite segments connect with each other; it can also be considered as the terminal end of one of these neurites. Because of this structure, all the influences, i.e., signal intensity and radius scale variations, from direct-connect segments should be considered. In our optimization model, we used simulation data instead of real datasets to eliminate the influence to some extent. Furthermore, we achieved an average branch point position by iteratively running the model, which will provide a more robust result.

The real skeleton of a tubular structure generally refers to its centerline. With automatic algorithms or even manual edit, it is impossible to completely obtain the real skeleton of a tubular structure. This is because no standard definition is available to characterize the real skeleton in images of tubular structures. Similarly, it is also hard to define the optimal skeleton of a tubular structure. In our model, the optimized skeleton refers to a solution to the designed optimization problems in which the centerline points are shifted to depict the actual neurite morphology while their signal intensities are maintained at a local maximum, as far as possible. Thus, the optimized skeleton is usually closer to the real skeleton, compared to the initial skeleton provided by other algorithms. Based on these assumptions, the initial skeletons acquired from different tracing algorithms can be optimized and lead to similar results in our model (Figure 6). This indicates that our detection method is compatible with other tracing algorithms, provides consistent results, and benefits the following analysis.

It is worth noting that our optimization model has been limited by some cases. Our model focuses on optimizing the position of the bifurcation point at present. For a multifurcation point, considering that it exists in few cases, our model has no special design to obtain its optimized position. In this case, a multifurcation can be divided into two or more bifurcations, and we can optimize the positions of these bifurcations to correct the position of this multifurcation point. However, the optimization accuracy will decrease because bifurcations interference with each other. In addition, the goal of our model is optimizing the skeleton of a neuron, and the skeleton data generated by the tracing methods is used as the input. The input skeleton data is required to be manually checked when high precision tracing results cannot be generated. This is because the errors in the input dataset like spurious links between traced neurites can decrease the performance of our model. Finally, our model used the signal in the neighboring region of traced neuron skeleton for the traced skeleton optimization. When neurons are densely distributed, the neighboring region usually contains morphological information from other neurons and this situation will negatively influence the optimization results. In the future, we will aim at this case and eliminate the interferential morphological information by segmenting and identifying the shape of the target neuron. Namely, our method will combine with the reconstructed shape for generating more accurately traced neuron skeleton.

In conclusion, we propose two models for optimizing the positions of intermediate points and branch points derived from an initial skeleton. These two models are based on the characteristics of neuronal morphology. Our results show that our method is effective when applied to various datasets including the MOST and BigNeuron datasets. The successful application of our method under different conditions demonstrates that it can generate a reconstructed skeleton that reflects reality better and will, therefore, have a positive impact on subsequent research.

## Data Availability

Our method is plugged into the open-source software GTree, which is available at https://github.com/GTreeSoftware/GTree/releases, including a user guide. We also applied some data and corresponding initial reconstructions used in our paper as examples for readers to try out; these can be download at https://github.com/GTreeSoftware/TEST_DATA/releases. Other data used in our paper can also be made available for downloading for readers who contact and ask for permission from the corresponding author.

## Author Contributions

SZ and TQ conceived the project. TQ and SL designed the model and wrote the manuscript. TQ and SL designed the algorithms. HK and YC wrote the software. CX, YC, and QH performed image analysis. QL, HG, and AL constructed the computing platform for large-scale image preprocessing. QL, HG, SZ, LF, and AL produced the dataset.

### Conflict of Interest Statement

The authors declare that the research was conducted in the absence of any commercial or financial relationships that could be construed as a potential conflict of interest.

## Supplementary Material

Figure S1 The distance between optimized and initial branch point as a function of square of sigma. Here, sigma refers to the Gaussian kernel width.

Figure S2 y-z and x-z perspective view displays of the reconstructions and original image stacks in Figure 6. **(A**_**1**_**,A**_**2**_) display the original image and reconstructions in Figure 6A from y-z and x-z views, respectively. The initial reconstructions from three tracing methods (red, yellow, and green, NeuroGPS-Tree, NeuronStudio and APP2, respectively) and their optimized reconstructions (purple, our model) are shown; **(B**_**1**_**,B**_**2**_**)** and **(C**_**1**_**,C**_**2**_**)** are the same image stacks shown in Figure 6B,C, respectively. The colors of these reconstructions depict the same meaning in **(A**_**1**_**,A**_**2**_**)**. The positions of the optimized branch point (solid dots) are given.
