# Supplementary figures and images for: Optimization of Traced Neuron Skeleton Using Lasso-Based Model
Source: Front Neuroanat. 2019 Feb 21;13:18. doi: 10.3389/fnana.2019.00018 (PMC6393391; doi:10.3389/fnana.2019.00018)

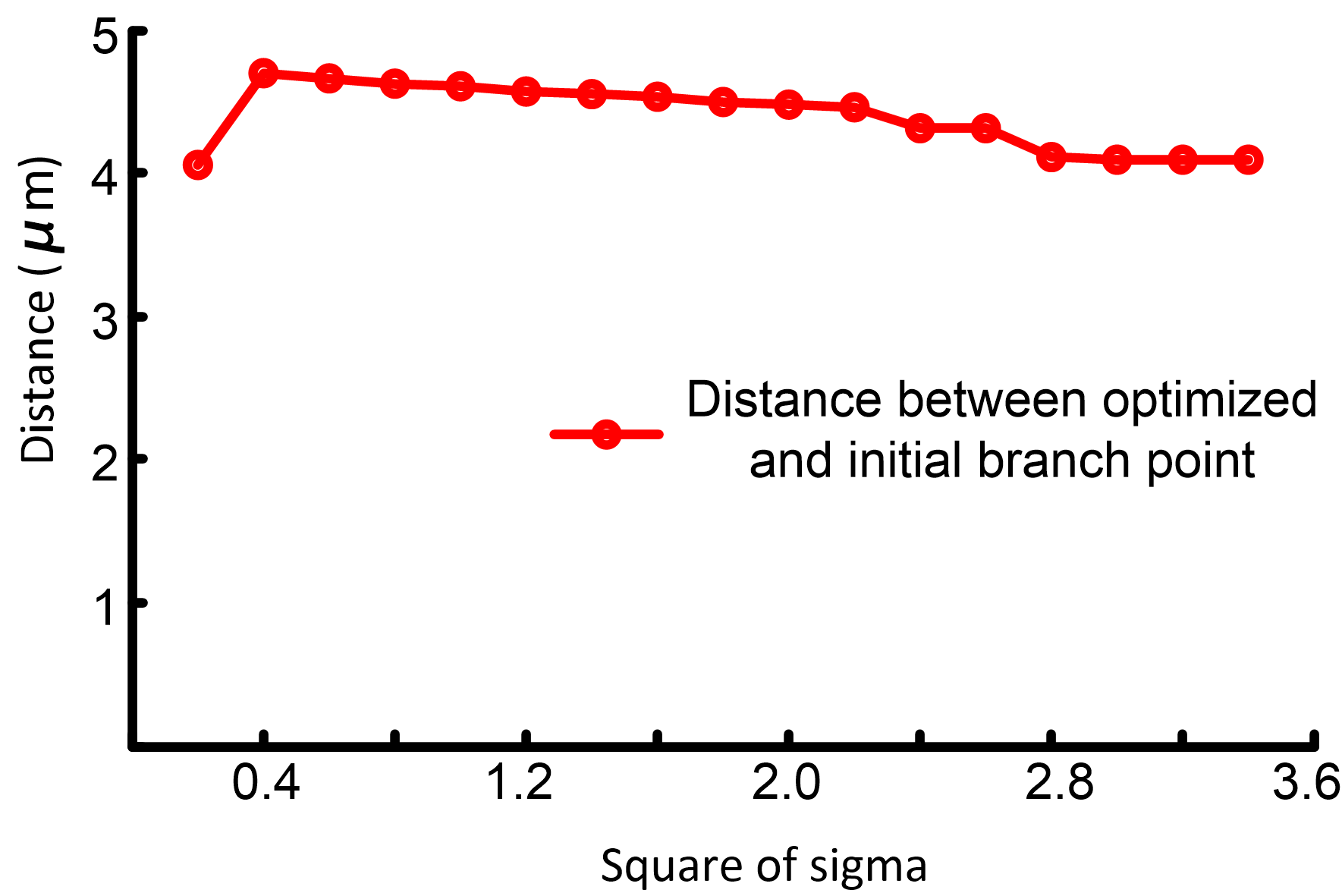

Supplement: Figure S1 — The distance between optimized and initial branch point as a function of square of sigma. Here, sigma refers to the Gaussian kernel width. [file image_1.tif]

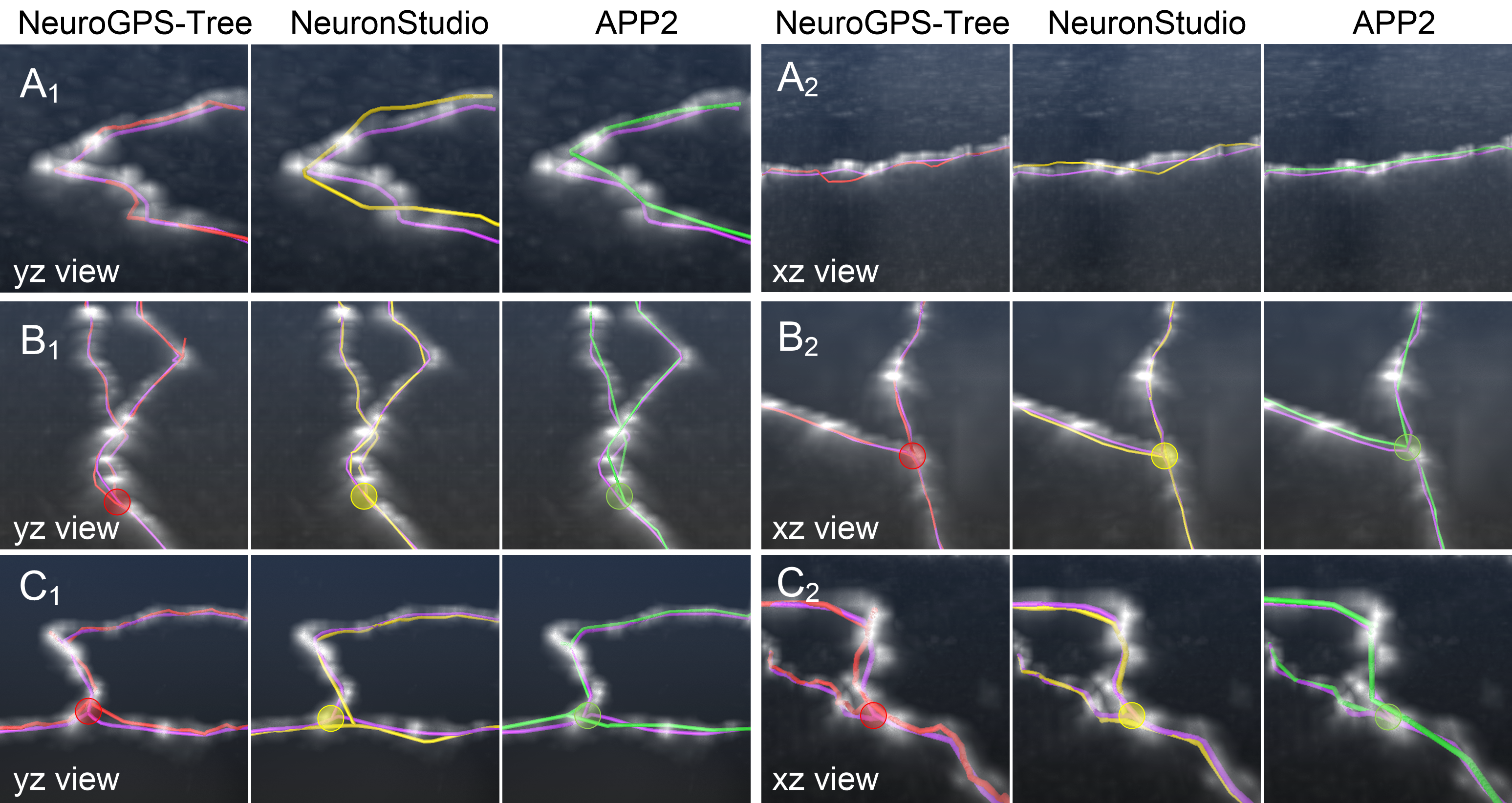

Supplement: Figure S2 — y-z and x-z perspective view displays of the reconstructions and original image stacks in Figure 6. (A1,A2) display the original image and reconstructions in Figure 6A from y-z and x-z views, respectively. The initial reconstructions from three tracing methods (red, yellow, and green, NeuroGPS-Tree, NeuronStudio and APP2, respectively) and their optimized reconstructions (purple, our model) are shown; (B1,B2) and (C1,C2) are the same image stacks shown in Figure 6B,C, respectively. The colors of these reconstructions depict the same meaning in (A1,A2). The positions of the optimized branch point (solid dots) are given. [file image_2.tif]
